# Hepatic flexure breakdown strategy with adaptive traction for endoscopic submucosal dissection

**DOI:** 10.1055/a-2598-4668

**Published:** 2025-06-18

**Authors:** Jean Grimaldi, Louis-Jean Masgnaux, Elena De Cristofaro, Timothée Wallenhorst, Jérôme Rivory, Jérémie Jacques, Mathieu Pioche

**Affiliations:** 1 Gastroenterology and Endoscopy Unit, Edouard Herriot Hospital, Hospices Civils de Lyon, Lyon, France; 2 Department of Systems Medicine, Gastroenterology and Endoscopy Unit, Tor Vergata University of Rome, Rome, Italy; 3 36684 Gastroenterology and Endoscopy Unit, Pontchaillou University Hospital, Rennes, France; 4 Gastroenterology and Endoscopy Unit, Dupuytren University Hospital, Limoges, France

Adaptive traction-assisted endoscopic submucosal dissection (ESD) has been shown to be effective in the treatment of difficult colonic lesions 1 2 3 4 . The hepatic flexure is one of the most challenging sites for colonic ESD, associated with longer resection times and more technical failures 5 .

We report here the case of a 68-year-old patient referred for ESD resection of a nongranular LST located on the mesenteric side of the hepatic flexure ( Video 1 ). The resection of this lesion was complicated by the access to the oral part of the lesion, which was very difficult due to the poor maneuverability of the endoscope ( Fig. 1 ). After making a circumferential incision, we positioned the adaptive multitraction device (ATRACT, Lyon, France) at the four cardinal points of the lesion ( Fig. 2 ). We then chose to attach the rubber band to the opposite colonic wall, not over the center of the lesion as it is usually done, but in the transverse colon downstream of the lesion. This allowed the hepatic flexure to be aligned with the transverse colon so that ESD was performed in a straight colon rather than an angled colon ( Fig. 3 ). The resection was R0 and without complications. The total procedure time was 40 minutes. The lesion was a high-grade dysplastic adenoma.

Hepatic flexure breakdown strategy for ESD.Video 1

**Fig. 1:**
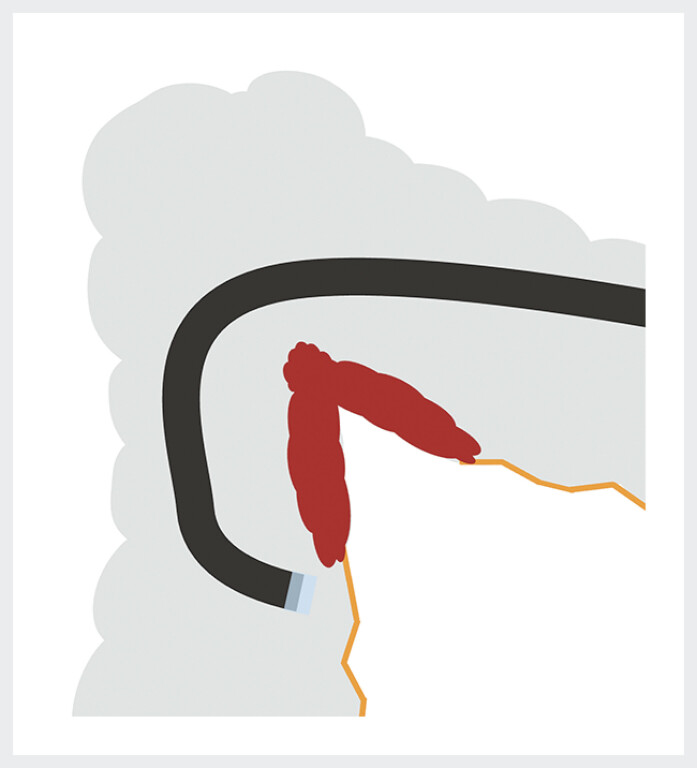
The lesion was located on the mesenteric side of the hepatic flexure with very difficult access to its cecal part due to poor maneuverability of the endoscope in the hepatic flexure.

**Fig. 2:**
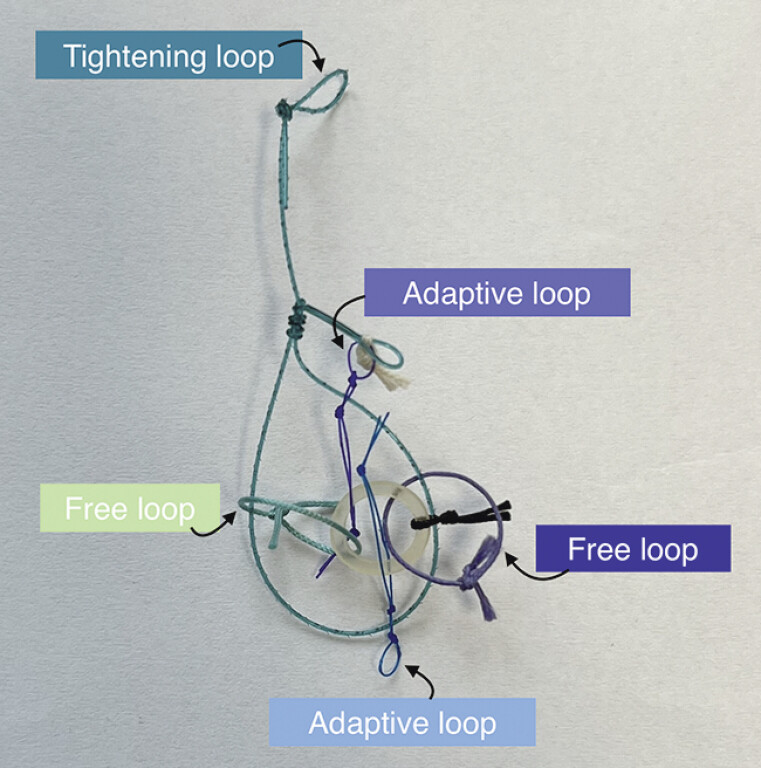
Adaptive multitraction device (ATRACT).

**Fig. 3:**
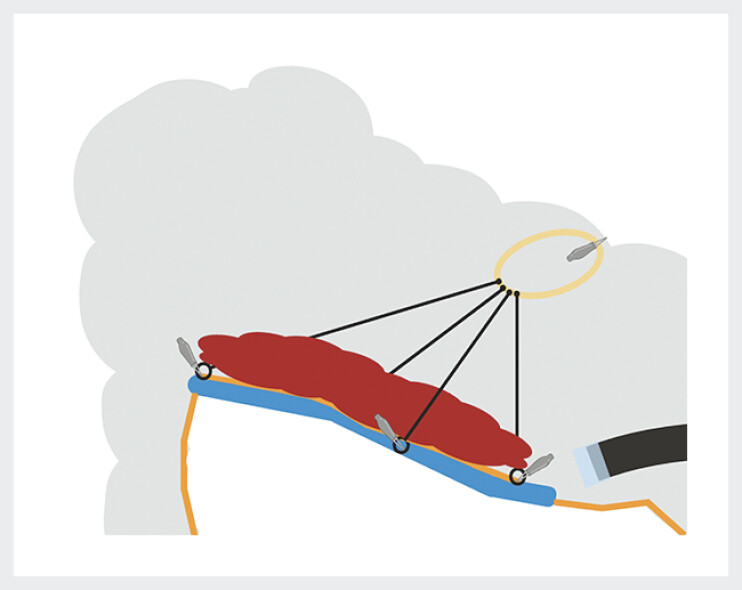
The rubber band of the ATRACT traction device was placed in the transverse colon downstream of the lesion, to align the hepatic flexure with the transverse colon to facilitate ESD.

This case illustrates the great interest of traction in difficult colonic lesions, not only to open the submucosal plane but also to modify the conformation of the colon when this makes resection more difficult. The development of computational modeling tools could help define optimal traction strategies for these difficult and unique lesions, whose resection strategy is still determined empirically today.

Endoscopy_UCTN_Code_TTT_1AQ_2AD_3AD

## Notes

Endoscopy E-Videos https://eref.thieme.de/e-videos
*E-Videos* is an open access online section of the journal *Endoscopy* , reporting on interesting cases and new techniques in gastroenterological endoscopy. All papers include a high-quality video and are published with a Creative Commons CC-BY license. Endoscopy E-Videos qualify for HINARI discounts and waivers and eligibility is automatically checked during the submission process. We grant 100% waivers to articles whose corresponding authors are based in Group A countries and 50% waivers to those who are based in Group B countries as classified by Research4Life (see: https://www.research4life.org/access/eligibility/ ). This section has its own submission website at https://mc.manuscriptcentral.com/e-videos .

## References

[1] Grimaldi J Masgnaux LJ Lafeuille P Endoscopic submucosal dissection with adaptive traction strategy: first prospective multicenter study (with video) Gastrointest Endosc 2024 100 517 523 38458261 10.1016/j.gie.2024.02.032

[2] Masgnaux LJ Grimaldi J Rivory J Endoscopic submucosal dissection assisted by adaptive traction: results of the first 54 procedures Endoscopy 2024 56 205 211 10.1055/a-2109-4350 37311544

[3] Grimaldi J Masgnaux LJ Rivory J Multipolar traction with adjustable force increases procedure speed during endoscopic submucosal dissection: the A-TRACT-4 traction device Endoscopy 2022 54 E1013 E1014 36002007 10.1055/a-1904-7666 PMC9736797

[4] Masgnaux LJ Grimaldi J Rostain F Endoscopic submucosal dissection of appendiceal lesions by using a novel adjustable traction device: A-TRACT-2 VideoGIE 2022 8 81 83 10.1016/j.vgie.2022.09.008 36820254 PMC9938289

[5] Li B Shi Q Xu EP Prediction of technically difficult endoscopic submucosal dissection for large superficial colorectal tumors: a novel clinical score model Gastrointest Endosc 2021 94 133 144 10.1016/j.gie.2020.11.012 33221323

